# What renewable energy future should we strive for? Assessing renewable energy utopias through Sci-Fi and normative energy ethics

**DOI:** 10.1186/s13705-025-00559-3

**Published:** 2026-01-13

**Authors:** Nynke van Uffelen, Daniel Wuebben, Giovanni Frigo, Roman Meinhold, Lorenzo Simone

**Affiliations:** 1 Faculty Technology, Policy and Management, Delft University of Technology (TU Delft), Jaffalaan 5, Delft, 2600 GA Delft, Netherlands; 2 School of Social Sciences and Humanities/Institute for Research in Technology, Universidad Pontificia Comillas, Madrid, Spain; 3 Institute for Technology Assessment and Systems Analysis, Karlsruhe Institute of Technology, Karlstraße, Karlsruhe, 76133 Germany; 4 Mahidol University International College, Mahidol University, 999 Buddha Monthon 4 Rd., Salaya, Nakhon Pathom, 73170 Thailand; 5 RWTH Aachen University, Willy-Brandt-Str.2, Bad Münstereifel, 53902 Germany

**Keywords:** Energy utopia, Just energy transition, Energy abundance, Energy sufficiency, Normative energy ethics, Energy justice

## Abstract

**Background:**

Socio-technical imaginaries, visions and utopias concerning energy and sustainability offer ideas about how the world should be. As such, they are normative endeavors that require a critical ethical assessment. However, normative assumptions about energy futures often remain implicit, thereby escaping critical scrutiny. This study combines science fiction and normative energy ethics to evaluate competing visions of renewable energy futures. We introduce a conceptual framework that distinguishes between the two main ways in which energy intersects with utopian futures: energy abundance and energy sufficiency. Next, we identify the ethical pros and cons of energy abundance and sufficiency as desirable future states, examining this through popular science fiction texts and normative energy ethics perspectives such as energy justice, virtue ethics, and critical theory of technology.

**Results:**

The vision of renewable energy abundance provides a very appealing prospect and can motivate different stakeholders to speed up the transition to a low-carbon energy system. However, striving towards such an energy utopia comes with several caveats. First, the idea of renewable energy abundance in the near future is dangerous because it is, so far, a technological illusion. Second, regional visions of energy abundance often neglect global and intergenerational energy justice considerations. Third, according to virtue ethics, pursuing energy abundance can be considered excessive, not virtuous and hence immoral. Fourth, energy abundance can lead to problematic forms of alienation and, therefore, dystopian versions of the good life. Utopias based on renewable energy and sufficiency aim to avoid these issues. Yet they face two additional problems that seem to hinder the adoption of energy sufficiency as the leading energy policy paradigm. First, there is a real danger that citizens would protest and slow down the energy transition if energy sufficiency were to be promoted by governments on a large scale. Second, in practice, the lines between energy sufficiency and abundance, and between energy needs and wants, remain unclear and highly contextual, leading to philosophical and practical problems.

**Conclusions:**

We propose distinguishing between two questions that may require different answers: Firstly, what kind of energy future do we, as a society, want? And what energy future should we strive for in our energy policies? Taking critiques of the pursuit of renewable energy abundance seriously, we conclude that we should resist the tendency to unquestioningly incorporate utopian ideas of renewable energy abundance into energy policies and technologies, despite the strong rhetorical appeal of abundance. This implies that the second concern regarding energy sufficiency — namely, its ambiguity, context dependency, and challenging measurement issues — should be addressed directly instead of being avoided. Energy policies must engage more explicitly with the normative assumptions underlying desirable energy futures, particularly with regard to sufficiency versus abundance.

## Background

Energy transitions are driven by assumptions about desired “futures”, which we consider as an umbrella term encompassing (large technological) visions, (sociotechnical) imaginaries [1], fantastic futures [2], and fantasies [3]. Desired socio-technical *energy* futures, like visions and scenarios, are narrative tools to shape the present [4]. They are often infused with utopian elements which supplement, influence, and shape the development of energy projects, policies, and cultures [5, 6]. Desired energy futures steer the energy transition, as they have the ability to align actors, collect resources towards common goals, and motivate action [7]. As a result, they might orientate the direction of technological innovation [8], provide justification for energy practices and behaviors, and steer energy and climate policies and decision-making [9]. Since the 2000s, social science research has increasingly focused on how futures are created and imagined and how they drive behavior, innovation processes, and policymaking [10]. It is often suggested that studying the visions of the future held by different actors can also help explain conflicts, energy transition delays, and differences in participation in the energy transition [6, 11–13].

Desired futures suggest ideas on how the world *should be*, and as such, they are normative endeavors. Because of their pervasive influence on climate policies and technological innovations, and thus on everyday human existence and societies, it is crucial to critically assess them from an ethical vantage point [5, 14, 15]. While previous research has focused on descriptively examining how utilities, promoters, and governments plan desired futures, there remains a critical gap in systematically identifying and ethically reflecting on the normative assumptions underpinning these desired energy futures. These ethical implications are increasingly pressing due to various environmental emergencies, including climate change, on the one hand, and demand for limited non-renewable resources required to build renewable energy technologies and systems, on the other. Energy futures should not be uncritically embedded in energy and climate policies, or in energy innovations. In this sense, the answer to the question ‘What energy future do we want?’ might not be the same as the answer to ‘What energy future should we strive for?’. Here, our audience is constituted by energy planners, policy makers, and the various stakeholders engaged in climate and energy politics. Our research question becomes: What desired futures can steer energy decision-making, and how can those futures be ethically evaluated? This question is pertinent because different stakeholders such as energy planners and policy makers may have competing conceptions about desired futures, and in such cases, it is unclear whose view should take precedence. Moreover, certain stakeholders may desire particular energy futures without understanding their broader implications for and impacts on other parts of society and other affected parties.

To pave the way towards ethical reflection on desired energy futures, we propose a conceptual framework distinguishing between two types of renewable energy futures, namely *renewable energy abundance* and *renewable energy sufficiency*. Similar concepts have already been explored by some scholars and are also present in popular science fiction. First, we argue that this distinction is a helpful conceptual tool to identify different renewable energy futures, and opens the door to critical reflection. Second, we flesh out risks and benefits of renewable energy utopias of abundance and sufficiency through (a) popular science-fiction novels and (b) normative energy ethics perspectives, such as energy justice, virtue ethics, and critical theory of technology, which further develop the critical potential within science-fiction novels by connecting them to examples in moral philosophy.

Fictional energy utopias display systems of energy conversion and consumption that concatenate with ideal, stable societies that offer affordable, reliable energy to most, if not all, of its members. While fictional energy utopias appear predominantly positive and anticipatory, they often also contain dystopian elements that may include catastrophic weather events, ecosystem collapse, mass extinctions, or violent uprisings. These writings can offer explicit and implicit blueprints for energy transitions and warnings of potential upheavals. Utopian and dystopian fiction portrays socio-technical alternatives and identifies the risks and benefits of different abundant or sufficient energy futures. Readers of these texts are prompted to visualize new energy forms, uses and relationships to energy [16–18]. In addition, when the normative energy ethics lens is applied to these science fiction texts, readers can gain new critical perspectives that can fertilize and influence public discourse. In short, more than simply imagining different energy systems and uses, we can imagine how to create more explicitly *ethical* energy futures.

The paper proceeds as follows. In the remainder of this section, we introduce the conceptual distinction between renewable energy utopias of abundance and sufficiency. Next, we draw on four novels of utopian science fiction to identify advantages and risks related to both ideals, and we explain our methodology for doing so in Sect. ''Methods''. In Sect. ''Results'', we present the results and in Sect. ''Discussion'', we critically discuss the energy futures presented in the novels through normative energy ethics perspectives. In Sect. ''Conclusions'', we conclude that while energy utopias of renewable energy abundance appear to be a valuable driver for renewable energy technologies and policies, they may negatively affect global justice, sustainability, and well-being. For these reasons, they should not be uncritically embedded in energy policies.

### Energy utopias of abundance and sufficiency

In this paper, we propose an analytical distinction between *renewable energy abundance* and *renewable energy sufficiency* as two types of ideal or desired energy futures. This distinction is inspired by Sci-Fi novels (see Sect. Results), historical studies on the development of energy systems, and utopian literature scholars. Already in 1999, De Geus [19] studied utopias in the history of political philosophy and introduced the distinction between *utopias of abundance* and *utopias of sufficiency*. The main difference pertains to “whether an ideal society should enjoy material abundance and luxury or be based on satisfaction and sufficiency” [19]. In this, utopias of sufficiency “posit ideal societies whose *raison d’etre* is the satisfaction of moderate human needs through harmonious social and ecological relations. These societies are characterized by simplicity and self-restraint rather than material abundance and overconsumption. They demonstrate how a high quality of life can be achieved through richness of community, sufficiency of goods, meaningful work coupled with significant leisure, and ecological integration” [20]. Moreover, in 2016, Schneider et al. introduced the concept of *energy utopias* as “a set of rhetorical appeals that positions a particular energy source as the key to providing a ‘good life’ that transcends the conflicts of environment, justice, and politics” [21]. We build on De Geus’ and Schneider’s contributions by linking *abundance* and *sufficiency* to renewable energy futures specifically. Thus, we distinguish between renewable energy utopias of *abundance* and *sufficiency*.

We are aware and acknowledge that “abundance” has been adopted by different voices with corresponding diverse uses, from degrowth [22, 23] to recent neo-liberal platforms [24]. Here, we define utopias of *renewable energy abundance* as envisioning an inexhaustible^1^ supply of renewable energy; in such worlds, worrying about issues such as energy affordability and access is no longer necessary, as enough clean energy exists to cover not only all human needs but also limitless wants. In this sense, these utopias may align with ideas of green growth, or ecomodernist interpretations [25] of sustainable development.

Desired futures of *renewable energy sufficiency* typically criticize (capitalistic) lifestyles of affluence and abundance and propose a minimalist (i.e., resource-conscious) lifestyle that is more than mere survivorship. Narratives of renewable energy sufficiency often imagine implementing an ecologically balanced renewable energy system that provides enough energy to fulfil only those needs that contribute to “true” well-being. As such, this energy utopia parallels the concept of *“buen vivir”* [26, 27] and degrowth.

Both futures of renewable energy abundance and energy sufficiency reflect utopian ideals that citizens can desire and strive towards. Moreover, both visions result in a different focus in policymaking. Futures of renewable energy abundance may drive policies stimulating technological innovations because realizing the satisfaction of all wants and needs implies more energy-consuming systems and, thus, more advanced renewable energy technologies. Moreover, policies that embed the idea of energy abundance will reject policies focused on degrowth and consuming less, as they envision maintaining or even increasing current consumption levels.

In visions of energy sufficiency, on the other hand, there are different socio-technical arrangements. For example, some authors proposed low-tech, minimalist imaginaries, while others embrace advanced and highly efficient energy systems. In both cases, energy sufficiency narratives tend to have different foci, namely on energy accessibility, eradicating energy poverty, tackling resource scarcity, and fulfilling basic needs. These visions of sufficiency are indebted to previous work by De Geus [19, 28] and Schneider [21]. In addition, as Michael Walzer explains in his *Spheres of Justice*, when it comes to ‘needs’, “It’s not having y, but only lacking x that is relevant” [29]. Policies that embed energy sufficiency would generally tend to focus on the needs of the most vulnerable, thus making sure no one is ‘lacking’ and that all actors have ‘enough’. Moreover, to ensure that vulnerable populations do not land below a certain capability threshold, energy policies that embed sufficiency will incentivize citizens to consume less, such as air travel and clothes.

To be clear, those who strive for renewable energy abundance are not opposed to all actors having sufficient available and affordable energy, and that everyone’s basic needs are met. On the contrary, its proponents may state that an abundance of renewable energy may ‘trickle down’ on the most vulnerable and as such, sufficiency for all will be ensured. In practice, both energy utopias may occasionally favor the same policies, instruments or means. So, the distinction between renewable energy utopias of abundance and sufficiency is a conceptual, analytical distinction, outlining different versions of the good life and a different set or hierarchy of values, which may lead to a different focus and set of priorities in the context of energy policy. We consider this distinction helpful and a promising critical lens for scrutinizing energy policies.

### A brief history of energy abundance

Utopian ideas about abundant energy resources can be traced back to processes of industrialization and (neo)colonialism in the late eighteenth century. As Western civilization began to industrialize, the demand for energy and raw materials increased, which led to a focus on locating, extracting, transporting, and converting energy sources into various energy services. In North America, colonizers profligately harvested lumber and animal skins, which they viewed as an “inexhaustible resource” [30]. In the early nineteenth century, coal mines, canal systems, and railroads intertwined to create “landscapes of energy abundance” that connected rural sites of extraction and urban sites of consumption [31]. After the turn of the twentieth century, electricity, oil, and natural gas began to replace coal. In the Global North, generally speaking, these “abundant and inexpensive” [30] fossil-fuel resources remain staples of energy use today.

Visions, imaginaries, ideals, and fantasies of ‘abundant’ energy resources are not restricted to the Global North. As Malone et al. argue [32], abundance has been a core feature of energy narratives in countries such as Brazil and Sweden. Moreover, ideals of abundance have not been restricted to fossil energy sources. As the environmental impacts of extractive practices became clearer and technological advancements gained pace by the mid-to-late twentieth century, there was a general shift towards other sources of energy, such as “too cheap to meter” nuclear power: “Abundant energy was seen as a prerequisite for permanent economic growth and nuclear power as a prerequisite for abundant energy” [33]. Similarly, the first utopian vision of plentiful hydrogen was formulated by Jules Verne in *The Mysterious Island* [34] and since then, hydrogen-fueled technologies have experienced several hypes, some of them of utopian character [7]. For example, proponents of hydrogen suggest that a decentralized hydrogen system would solve ecological problems and lead to equality and democratization of energy and power [35]. These developments have solidified the feeling that “as long as humans could access ever larger stocks of accumulated abundance, rapid growth can be maintained indefinitely” [31].

Given the modern and prevalent hope for energy abundance, scholars have recently argued that the abundance ideal is intrinsically linked to capitalism and liberalism, both of which are threatened as the ideals of abundance are challenged [36]. As such, it is unsurprising that ideals of energy abundance have also been formulated in relation to renewable energy. Linguistic expressions and passages that reflect these tendencies can be found in recent crucial public policies such as the European Green Deal and the USA Green New Deal. To illustrate, for example, Frans Timmermans, the former Executive Vice-President of the European Green Deal, claimed that “renewables are a cheap, clean, and potentially endless source of energy [.]” [37]. Relatedly, the ​​EU Green Deal is presented as a new growth strategy that aims to achieve climate neutrality and “transform the EU into a fair and prosperous society, with a modern, resource-efficient and competitive economy where there are no net emissions of greenhouse gases in 2050 and where economic growth is decoupled from resource use” [38]. In a similar direction, The USA Green New Deal envisions “massive growth in clean manufacturing in the United States and removing pollution and greenhouse gas emissions from manufacturing and industry as much as is technologically feasible, including by expanding renewable energy manufacturing and investing in existing manufacturing and industry” [39]. Therefore, ideals of energy abundance still shimmer through renewable energy policies nowadays, and they appear to retain an *eutopian* appeal for many regardless if they remain unattainable.

### A brief history of energy sufficiency

In the 1970s, the fictional treatment of energy abundance and energy sufficiency formed part of a broader discourse on the energy crisis and increased efforts to conserve resources and become more energy-efficient [40]. The Oil Embargo of 1973 created a sudden scarcity of fossil fuels and soaring costs, both of which influenced public discourse about energy efficiency. By 1979, the nuclear accident at Three Mile Island and Second Oil Crisis sparked by the Iranian Revolution further reinforced a growing awareness of the risks posed by the seemingly endless supply of and demand for energy. In the intervening decades, appeals to energy efficiency, energy independence, and ‘peak oil’ have continued to shape energy discourses. Not abundance, but sufficiency, including the notion of efficiency is implied in the “Brundtland Report” Sustainable Development definition [41]. Indeed, that definition has an emphasis on “energy efficiency” and “energy savings” to secure future human development within ecological boundaries. The relationship between “sufficiency” and “efficiency” is interesting as they seem often complementary, particularly in the case of techno-optimistic energy futures. Bourliaguet, for example, has recently showed how energy sufficiency is “becoming the fourth pillar of the French energy transition, alongside energy efficiency, nuclear power generation and renewable energy” [42]. However, more generally, recent research has shown that, since the 1990s, corporate understandings of sustainable development permitted only gradual improvements in sustainability practices while failing to address the urgent necessity of implementing more aggressive measures to prevent increasing emissions and environmental degradation [43]. This suggests that there are essential tensions between the goals of capitalism, energy transitions, and the demands of strong sustainability. While the notion of “efficiency” seems to be a shared motive, that of “sufficiency” is much more contested as it relates to different understandings of “how much is enough” for certain living standards and levels of wellbeing, which tend to be considered very diverse depending on the stakeholders and spatio-temporal contexts.

In the broader film and literary culture, dystopian films such as *Mad Max* (1979) and postmodern novels such as *Gravity’s Rainbow* (1973) reflected a growing awareness of planetary limits and increased skepticism of government-funded research and development programs. In 1974, Ivan Illich exposed the myth of abundance in an essay titled “Energy and Equity”, which argues against the notion that “clean and abundant energy is the panacea for social ills” [44]. He goes on to explain that “Even if non-polluting power were feasible and abundant, the use of energy on a massive scale acts on society like a drug that is physically harmless but psychically enslaving” [44]. In this context, it is unsurprising that, in the 1970s, Sci-Fi authors such as Le Guinn and Callenbach were able to attract large audiences with narratives undercutting the myth of energy abundance. Indeed, previous research on energy in fiction has shown how popular literature asks readers to question myths of abundance. In “Literature and Energy Futures” [45], Szeman claims that science fiction can “shake us out of our faith in surplus” (p. 325). Similarly, Patricia Yaeger explains that in fictional representations, fuels like wood, coal, and gasoline reveal the social distinctions of the “system of mythic abundance not available to the energy worker who lives in carnal exhaustion” [46].

### Sufficiency and abundance as interpretative tools

The distinction between energy sufficiency and abundance as desired renewable energy futures, we argue, can be used as a torchlight to study the implicit normative ideals embedded in policy documents and energy systems. The typology can also foster self-reflection about the future we, as citizens, and in our professions, want to achieve. We assume that both ideals exist in the empirical world and that they may clash and cause conflict in processes of technological innovations and design, policymaking, behavior choices, and activism. Moreover, based on the history of our energy system, we may assume that the dominant envisioned future in industrialized nations with high energy consumption (i.e. most societies in the Global North) is that of abundance. In general, scenarios with significantly higher energy demand seem to outnumber scenarios with lower energy demand. This is reflected, for example, in the IPCC’s 6th Assessment Report scenario database, where most pathways project an increase in energy demand [47]. In places, the authors attribute this increasing demand, in part, to expanding universal access to modern energy services, which would increase demand and global GHG emissions and improve living standards. The growing demand is also possible due to a “digital transformation,” which they warn could “increase energy demand, exacerbate inequities and the concentration of power, leaving developing economies with less access to digital technologies behind, raise ethical issues, reduce labor demand and compromise citizens’ welfare” [47]. As we will discuss later, the distinction between ideals of energy abundance and sufficiency allows for critical reflection and paves the way for normative assessments.

## Methods

Since our aim is to explore critical perspectives on both renewable energy abundance and energy sufficiency, we selected Sci-Fi novels that explicitly and critically address one or both utopian ideas. Regarding relevant novels, we made an initial search for science-fiction works that meet the following criteria: Science-fiction novels.

1. Written in the English language and published after the second half of the twentieth century, as this period restriction ensures that the selected works reflect modern energy infrastructures of nuclear power systems and the post-World War II expansion of electricity networks in North America and Europe;
2. That depict either an energy dystopia, in which energy is scarce and/or used to wield totalitarian power of populations, or an energy utopia, which we defined as rendering (almost) ideal systems of energy sourcing, conversion, planning, distribution, utilization and its related recycling or waste management;
3. Recognizing that energy systems have significant environmental impacts; and.
4. That directly and critically engage with ideals of renewable energy sufficiency and/or abundance and show how these ideals intersect with social norms and government policies.

The selection process involved snowballing within our scholarly network and proactive searches based on academic research in the fields of literature, science, and environment. As such, we do not claim to have included all relevant novels, and the conclusions in this paper are not meant to be generalizable to all Sci-Fi novels in this genre. Rather, we aimed to explore a range of different ethical assessments related to renewable energy sufficiency and abundance.

**Table 1:** Novels considered for the analysis

Title	Author	Year
*The Island*	Aldous Huxley	1962
*We*,* In Some Strange Power’s Employ*,* Move on a Rigorous Line*	Samuel Delany	1968
*The Dispossessed*	Ursula Le Guin	1974
*Ecotopia*	Ernest Callenbach	1975
*The New Atlantis*	Ursula Le Guin	1975
*Station Eleven*	Emily St. John Mandel	2014
*American War*	Omar El Akkad	2017
*Ministry for the Future*	Kim Stanley Robinson	2020

Our search initially identified eight novels that seemingly fit all four criteria (see Table 1). However, after a closer read to assess their explicit engagement with the fourth criterion related to energy sufficiency and abundance, we determined that four novels met al.l four criteria (see Table 2): Ursula Le Guin’s *The Dispossessed* (1974) [48] and “The New Atlantis” (1975) [49], Ernest Callenbach’s *Ecotopia* (1975) [50], and Kim Stanley Robinson’s *The Ministry for the Future* (2020) [51]. Each of these novels describes utopian and/or dystopian societies and directly engages critically with themes of renewable energy sufficiency and abundance. We acknowledge that additional novels may offer further arguments that could supplement our analysis.

**Table 2:** Final selection of four fictional energy utopias that explicitly critically evaluate strong themes of energy sufficiency and/or abundance

Selected novel	Category	Type of utopia
Ursula Le Guin, *The Dispossessed* (1974)	Soft far ‘anarchist’ utopia	Sufficiency
Ursula Le Guin, *The New Atlantis* (1975)	Soft near Dystopia	Abundance
Ernest Callenbach, *Ecotopia* (1975)	Hard near Eu-topia	Sufficiency
Kim S. Robinson, *The Ministry for the Future* (2020)	Hard near utopia with dystopian elements	Sufficiency

The four novels represent a mix of near and far utopias, as well as soft and hard science fiction (Table 2). Near fictional utopias refer to places or events that readers could imagine occurring within the current or next generation (approximately the next 20–50 years), while far utopias typically occur in a distant future that is several generations or thousands of years away. Near (e)u-topias are of interest in this article because they take into account climate facts as well as technological developments related to energy cycles (i.e., energy sourcing, generation, consumption, waste disposal, or recycling). Another relevant distinction that is often used in literary studies is that between hard and soft fiction [52, 53]. Soft science fiction has a looser relationship with scientific accuracy, whereas hard science fiction takes its scientific base more seriously. Soft utopias are also relevant, as their less strict application of scientific facts allows for the imaginative exploration of societal potentials and dangers related to climate and energy systems. We analyzed the four novels by using interpretivist methods including a non-systematic and discussion-based reading of the novels among all co-authors.

As our results will show, *Ecotopia* and *The Ministry for the Future* are two near-hard Sci-Fi novels that portray energy technologies that have been or are projected to be implemented in the near future. In contrast, the two works by Ursula K. Le Guin, *The Dispossessed* and *The New Atlantis*, explore more speculative technologies and social configurations. While The New Atlantis sketches a dystopian world in which renewable energy abundance plays a more positive role, the remaining three novels primarily lean toward sufficiency-oriented narratives. As such, the four novels highlight both opportunities and challenges of moving beyond an abundance paradigm.

In the next section, we present for each novel the main narrative, with a focus on whether the novel discusses ideas of renewable energy abundance or sufficiency and whether these futures are described as ideas to strive toward or as less-than-ideal futures and why.

## Results

### Le guin’s the dispossessed (1974)

In Le Guin’s *The Dispossessed* (1974), the exploitation of new energy sources, the development of new energy technologies, and the dangers of energy abundance are central. This far-soft utopia offers a critical perspective on present-day energy utopias as it juxtaposes three distinct societies thousands of years into the future in which abundance and sufficiency play specific roles. Le Guin imagined her novel as an “anarchist” yet “ambiguous” utopia due to the conflicts between the lifestyles and energy uses in these distinct societies.

The first, Annares, is the anarchist satellite moon colony inhabited by the exiled Odonians. These followers of Odo, philosopher of sufficiency, consider their previously uninhabited moon as the “Eden of Annares”, but the actual landscape is “dry, cold, and windy, and the rest of the planet was worse.” The scarcity requires the Odonians to fit themselves, with great care and risk, into a “narrow ecology” (p. 155). Still, the Annaresti seem to have achieved a good life, in part through their practice of energy sufficiency, as exemplified by the following passage:

“There was no artificial lighting provided from an hour before sunrise to an hour after sunset. No heat was furnished when the outside temperature went above 55 Fahrenheit. It was not that Abbenay was short of power, not with her wind turbines and the earth temperature differential generators used for heating; but the principle of organic economy was too essential to the functioning of the society not to affect ethics and aesthetics profoundly. ‘Excess is excrement,’ Odo wrote in the *Analogy*. ‘Excrement retained in the body is a poison.’” (p. 84).

In contrast, in the capitalist nation of A-Io on the planet Urras, the public markets are filled with products that are “either useless to begin with or ornamented so as to disguise its use; acres of luxuries, acres of excrement” (p. 110). From the perspective of the protagonist who travels from Annares to Urras, the gluttonous overcompensation of goods and energy reflects a social obesity that makes him nauseous. Of course, the A-Io governing body believes that they are acting sustainably. They deploy taxes and regulations on “luxuries” such as private vehicles, reasoning that without such punitive measures, the “public would tend to drain irreplaceable natural resources or to foul the environment with waste products” (p. 70). From the government’s perspective, without regulations, the people of A-lo would destroy the commons; from the Annaresti’s view, the commons have already been replaced by consumerism.

The third social arrangement of *The Dispossessed* exists on the future planet Earth, which has cycled from over-consumption and overshoot of earth’s planetary boundaries to collapse. Readers learn that Earth had been “spoiled by the human species” who “controlled neither their appetite nor violence” (p. 286). Thus, in this fictional future, the earth is signified by the “total control over the use of every acre of land, every scrap of metal, every ounce of fuel. Total rationing, birth control, euthanasia, universal conscription to the labor force […] absolute regimentation […] towards the goal of racial survival” (p. 287). Earth, from the viewpoint of the societies who have escaped, is a regimented dystopia in which living is reduced to surviving.

Le Guin’s Sci-Fi narrative explicitly condemns abundance, equating it with excrements or toxic substances. The novel leads readers to wonder: Is the primary obstacle to creating societies of sufficiency a limited amount of resources (e.g., energy scarcity), or the inability to control human desires? Overall, *The Dispossessed* presents the ideology of abundance is toxic and, as it spreads through society, it depletes resources and creates unstable social structures. People living in abundance lead unhealthy and unsatisfied lives. People living in harsh environmental conditions with energy sufficiency are more conscious of their consumption behaviors and are, for a time, happier.

### Le guin’s “the new Atlantis”

Ursula Le Guin’s 1975 near soft Sci-Fi novella “The New Atlantis” adopts the title of Bacon’s famous text and transforms his notion of a utopian island in the Pacific into a vision of modern environmental and social conflict. It describes a tension between renewable energy technologies and utopias of renewable energy abundance. In this dystopia, pollution has produced ecological collapse across North America, rising sea levels, and frequent earthquakes. The protagonists are under the authoritarian control of the government and suffer from chronic food and energy shortages. The authoritarian government’s power system, located across the Pacific Northwest, looks like an “electrified fence all around the forest to keep out unauthorized persons” (p.60). Readers are not privy the exact characteristic of the government’s power system, but it is implied that it is one of energy scarcity in which limited resources are retained by those in power leading most of the population to live in energy poverty.

Despite the overall dystopian setting, the protagonists imagine a better society in which there is renewable energy abundance. This idea is sparked by a “sun tap,” a cheap and simple device that collects and stores solar energy through “direct energy conversion.” The device is so powerful that “ten minutes of sunlight will power an apartment complex like ours, heat and lights and elevators and all, for twenty-four hours; and no pollution, particulate, thermal, or radioactive” (p. 74). Developed by renegade scientists, the sun tap will “completely decentralize industry and agriculture” by allowing abundant, free electricity to topple existing power structures and revolutionize political power towards more energy democracy. The government built fences around the forest to keep the people out; when the people regain power they will “build an electrified fence outside around the White House” to keep the authorized persons inside.

This soft-near energy utopia is carried by the ideal of a revolutionary technology that produces energy abundance for all. This distinguishes Le Guin’s novella and the other utopias we analyze from its namesake, Francis Bacon’s *New Atlantis* (1627). While Bacon’s descriptions of this advanced society occupying a fertile island in the Pacific Ocean reveal the “utopian promise of modern science,” [54]. Bacon does not explicitly state how scientific knowledge improves the lives of all the island’s ordinary citizens [55]. If there is energy abundance in Bacon’s *New Atlantis*, it seems likely that it will be reserved for the island’s elite. Therefore, while in Le Guin’s novella, the news of a new utopian island emerging in the Pacific Ocean seems to offer the protagonists hope of escape to a new geographic space, the imagined solar technology and its “unlimited” energy. One might assume that the solar tap would create abundance and provide a different kind of energy future, in which energy can “serve life” instead of capital, yet Le Guin leaves this possibility open and a direct connection between the new technology and the emerging island is never made.

### Callenbach’s ecotopia

Callenbach’s *Ecotopia* (1975) hard science fiction novel offers clear visions of how existing technologies might be deployed in innovative ways. In *Ecotopia*, a violent civil war results in the foundation of an economically self-sufficient and independent nation-state on the West Coast of North America. Ecotopia is an eco-social eu-topia, a positive or “good” (Greek: “eu”) utopia, that exemplifies “the possibility that a society could live in harmony with its environment while continuing to utilize many of the advances made through modern technology” [57, also quoted in 58]. In *Ecotopia*, energy technologies are central to this transformation. Power is generated decentralized and consumed locally (p. 102). Electricity is converted almost exclusively from photovoltaic, hydro, and ocean thermal energy conversion (OTEC). The transition from a fossil-based system to a circular economy supplemented by biogas produced as a by-product from sewage recycling units and used for private household food preparation and heating (p. 104) as well as low-tech reliable solutions such as homemade water wheels (p. 105). Ecotopian energy ethics is grounded by the ecosystemic awareness that the whole of society is a subset of the ecosystem [58]. Many newly implemented energy conversion systems in ecotopia utilize biomimicry or “homeotechnology” [59], meaning technology that either mimics biological mechanisms or, in a wider sense, follows processes already existing in the natural ecosystem, using nature itself as a guideline. Energy production simply follows the natural flow or movements of water, wind, tide, and temperature variations [58]. In *Ecotopia*, “houses tend to be abominably ill-lit” (p. 124); however, the energy *needs* of all citizens, not necessarily the wants of specific stakeholders, are met sufficiently. Here, energy utilization follows the ideals of fairness and sustainability instead of energy abundance. This is partly due to the high electricity cost, which reflects the real costs (p. 18) and internalizes the negative external effects of energy conversion. Another reason for restricting energy consumption is their environmental awareness, already taught early on in schools, and the cultural norm of limiting consumption to the necessary minimum. In this novel, sufficiency is not portrayed as a dystopian scenario but as a positive value, a mindset, and a lifestyle that contributes to individual and societal well-being.

### Robinson’s the ministry for the future

Robinson’s *The Ministry for the Future is* a near-hard climate fiction that features energy eu-topian elements and dystopian climate scenarios. The novel begins with a horrific heatwave in the Indian region of Uttar Pradesh during which the wet-bulb temperature surpasses 35 degrees Celsius for days. As the punishing heat wave continues and electrical grids fail, the masses are unable to keep cool even when submerged in lakes and rivers. Approximately do million people die of hyperthermia. Approximately 20 million people die. The narrative then loosely follows two characters. The first is Frank May, an American activist who was the sole survivor of the Indian heat wave, who became radicalized and began to battle the ideology of energy abundance by killing those who lived by it (p. 74, p. 93). He sympathizes with eco-terrorists who assassinate CEOs of fossil fuel companies, bomb passenger aircrafts and kill cows intended for meat production. The second protagonist is Mary Murphy, who leads the eponymous Ministry for the Future, a UN Agency working to implement international climate goals. Murphy’s greatest achievement is the worldwide implementation of the carbon coin. As major economies – China, the United States, and the European Union – adopt the coin, clean energy technology systems supplement the trading of carbon coins for climate change mitigation. Meanwhile, the rest of the Ministry supports massive geoengineering efforts (such as pumping water to help freeze ice caps and the development of rewilding corridors) and innovative ships and airships that use wind and solar technologies to convert more electricity than needed for their own transportation.

This novel proclaims that renewable energy abundance is a dangerous myth and that ignoring this fact causes global climate disasters. By the end of the novel, the massive deployment of renewable energy conversion technologies helps decarbonize economies and achieve the “utopia” of a livable planet. Yet within this utopia, a true abundance of energy does not exist. Instead, “all the necessities for a good life are abundant enough that everyone alive could have them.” The ideal of sufficiency here is conveyed as “enough”: “Enough should be a human right, a floor below which no one can fall; also a ceiling above which no one can rise. Enough is as good as a feast—or better. Arranging this situation is left as an exercise for the reader.” The exercise for us, as readers and scholars, is to show how this “enough” acts as an eutopia of sufficiency and how the ethical importance of sufficiency can be leveraged through international collaboration. Unlike the other three books in which the utopian society is physically and politically separated from other societies, *The Ministry for the Future* envisions a global transformation towards sufficiency. Robinson does not, however, explicitly address all implications of high-tech development, extraction of raw materials or the energy demand of the carbon coin, its computing requirements and corresponding data centers.

By positioning *The Ministry for the Future* within broader sustainability and climate discourse, one can associate the fictional heatwave with the real-life hurricanes that have recently ravaged North and Central America and the massive floods that swept through China, Thailand, and Germany. Furthermore, most readers of *The Ministry for the Future* will be aware of urgent appeals for action emerging from the recent UN Climate Change Conferences of 2019 in Madrid and 2021 in Glasgow, where Robinson was an invited guest speaker.

### Summary: an overwhelming critique of energy abundance

In all four texts, utopias of renewable energy abundance are unpacked, deconstructed, and praised or critiqued as an ideology. The latter is done in two main ways. On the one hand, the authors show that renewable energy abundance is a dangerous myth that often precedes social and environmental collapse (dystopia), forcing societies into a lifestyle of renewable energy sufficiency or worse. On the other hand, in the majority of the works analyzed here, renewable energy sufficiency is pursued as a societal ideal that improves the quality of life for society and individuals, with *The New Atlantis* being the exception, as renewable energy abundance remains the dream of the good life, motivating actors towards change. Therefore, while in Le Guin’s novella, the news of a new utopian island emerging in the Pacific Ocean seems to offer the protagonists hope of escape to a new geographic space, but this possibility is not directly linked to the imagined solar technology. One might assume that such the solar tap would create abundance and provide a different kind of energy future, in which energy can “serve life” instead of capital, yet Le Guin leaves this possibility open and a direct connection between the new technology and emerging island is never made.

## Discussion

The previous section shows that Sci-Fi provides preliminary ethical perspectives on both renewable energy abundance and sufficiency as desired futures. In this section, we elaborate on and expand on these ethical assessments through various normative energy ethics perspectives. Analysis of these fictional texts provides preliminary insights about visions of energy abundance and sufficiency. Here, we elaborate and expand on the ethical dimensions by applying normative energy ethics perspectives. In Sect. ''Benefits and limits of utopias of abundance'', we review the opportunities and risks of renewable energy abundance ideals for the energy transition [44, 60, 61]. In Sect. "The ambiguity of energy sufficiency", we reflect more thoroughly on the notion of renewable energy sufficiency and how it raises fundamental questions about the relationships among energy consumption, justice, and well-being. Section ''Outlook: What future should we strive for? '' balances both perspectives.

### Benefits and limits of utopias of abundance

Renewable energy utopias of abundance paint pictures of unlimited, green, guilt-free energy systems that allow users to maintain or even increase current levels of energy consumption for work and leisure. In his apology for solar punk, Andrew Hudson suggests that “abundance is a paradigm that breaks people out of the zero-sum thinking that makes poverty and deprivation seem unavoidable” [62]. Indeed, energy abundance provides a very appealing picture, and it can motivate different stakeholders to accelerate the transition towards a low-carbon energy system [7, 63]. For example, financiers are incentivized to invest in renewable energy, energy users are encouraged to buy solar panels and energy-efficient household equipment, and politicians can utilize hopeful images of the future to counter citizens’ fear of losing energy access and affordability.

However, as the Sci-Fi novels show, energy utopias of abundance come with several caveats that can be further articulated through normative energy ethics perspectives. First, renewable energy abundance in the near future is a dangerous illusion from socio-technical and scientific attainability standpoints. Second, regional visions of energy abundance frequently neglect global energy justice. Third, pursuing energy abundance can be considered immoral according to virtue ethics. Fourth, from a critical theory of technology perspective, energy abundance can lead to alienation and, therefore, to dystopian versions of the good life. The first two critiques pertain to embedding energy abundance as a desired future in energy policies, while the final two critiques relate to the ethical nature of the utopia itself. The four limitations of energy abundance are further illustrated in the remainder of this section.

First, renewable energy abundance can be considered a techno-utopian illusion. The visions of technological fixes seem to sidestep some of the ethical conundrums and ignore planetary boundaries. In *The Dispossessed*, abundance is portrayed as an ideology that depletes resources as it spreads through society. Similarly, *The Ministry for the Future* illustrates that failing to acknowledge this myth leads to global climate disasters. These ideas are not so far-fetched because, firstly, a complete circular economy with closed loops of resources and energy remains impossible to achieve. Due to the material implications of the second law of thermodynamics, recycling processes always require energy and are always incomplete due to entropy, generating waste and side-products [64, 65]. Therefore, “100% complete nature-economy-nature-economy etc. cycles will not be achieved any time soon, perhaps never” [65], also because they are typically affected by rebound effects (e.g., Jevons’ paradox). Korhonen et al. argue that a circular economy, “like all material and energy using processes, [.] too will ultimately lead to unsustainable levels of resource depletion, pollution and waste generation if the growth of the physical scale of the total economic system is not checked” [65]. Moreover, many technology-critical elements needed for energy technologies are scarce and their disposal can be environmentally harmful, which is typically ignored in utopias of abundance. Ignoring the illusionary character of renewable energy utopias of abundance implies hoping for quick “technofixes” or even a technological miracle. In this sense, embracing the notion of renewable energy abundance together with technological optimism resonates with the ecomodernist call for a high-energy planet [66] where humans are eventually able to combine sustainability and high standards of living, or capable of decoupling economic growth from environmental impacts [25]. However, if technological solutions do not present themselves (on time) and current consumption levels are maintained, humanity risks an even more rapid exploitation of resources, thus crossing planetary limits. In this view, by concealing planetary limits, utopias of abundance are ideologies that justify current consumption practices for the privileged.

Second, utopias of renewable energy abundance embedded in energy and climate policies can produce global inter- and intragenerational injustices. This is especially relevant for regional or national energy policies striving for renewable energy abundance. By investing in technologies while ignoring planetary limits beyond one’s jurisdiction, major social and material inequalities on a global level might be overlooked, such as insufficient access to resources or human rights violations within the global supply chain [67, 68]. Such global injustices occur in relation to hydrogen [69] and renewable energy infrastructures [70–72]. Relatedly, pursuing energy abundance as an ideal for the current generation can be intergenerationally unfair, as extracting limited resources today may discount future generations’ needs [73], with nuclear energy as a case-in-point [74]. In sum, striving for abundant renewable energy within a specific region and time can conceal injustices elsewhere. Nonetheless, abundance can be envisioned in different ways. In Callenbach’s *Ecotopia*, for example, there is a society built on generosity, cooperation, and an “economy of biological abundance” that prioritizes ecological stability over growth. Yet, even the Ecotopians acknowledge the limits of their vision, relying on some resource extraction and international trade to maintain their current systems while aspiring toward the “stable-state life systems which are our [their] fundamental ecological and political goal.” Indeed, for Ecotopians, circularity remains an ongoing pursuit, a challenge, rather than a fully realized achievement. Another and much more controversial issue related to justice emerges in the Ministry for the Future where in Chap. 25 the protagonists’ exchange mention that some killings might bring about justice:

You’ve been killing people?

“Yes.” He swallowed hard, thinking about it. “I’ll get caught.

eventually.”

Why do you do it?

I want justice!

Vigilante justice is usually just revenge.

He waved her away. “Revenge would be okay. But more importantly, I want to help to stop it happening again. The heat wave, and things like it.”

We all want that.

His face went red again. Choked voice again: “Then you need to do more.”

Third, energy abundance does not necessarily represent a precondition for achieving the good life. This idea was illustrated in *The Dispossessed*, where people living within energy abundance suffer from “social obesity”, in other words, they lead unhealthy and unsatisfied lives. More specifically, virtue-based ethical theories frequently define *bad* or *evil* as excess or limitlessness. For example, Aristotle’s virtue ethics account locates virtues between two extremes which are both seen as excesses or vices [75]. Acting virtuously means selecting a balanced option between forms of excess or deprivation, both seen as the problematic “extremes” or “vices”. In this view, excess is detrimental for virtue development and true flourishing of both humans and society, both of which are necessary conditions for happiness (eudaimonia). Besides the classic Aristotle, others have supported the existence of connections between form of sobriety, sufficiency, and minimalism to the exercise of a virtuous life. The work of Ivan Illich, for example, is often making these connections also in the context of energy [44, 76]. In the field of ethics of technology, for example, A. Borgmann stressed the importance of “focal practices” that are often linked to a more simple lifestyle [77]. Applying these reflections to our topic, energy sufficiency would represent a balance between scarcity and abundance, distinguishing between needs and wants, resembling the virtue of frugality. This virtue requires continual repetition in various contexts so that it becomes habitual. In other words, it is virtuous to turn the lights off when leaving the room, even if you have solar panels on your roof. Practicing frugality together with other virtues would then provide the condition for a virtuous life, which is the basis for achieving well-being or happiness (eudaimonia). Conversely, abundance is a vice that hinders a good life.

Fourth, critical theory of technology offers several avenues of critique that may be applied to energy utopias of abundance. Critical theorist Rahel Jaeggi argued that there are functionalist, moral, and ethical critiques of capitalism: “The functionalist argumentative strategy holds that capitalism is intrinsically dysfunctional and crisis-prone; the moral or justice-oriented mode of argument asserts that capitalism is morally wrong, unjust, or based on exploitation; and finally, the ethical critique contends that a life shaped by capitalism is a bad, impoverished, meaningless, or alienated life” [78]. The functionalist critique corresponds to our first point about the illusion of energy abundance; the moral or justice critiques relate to our points on virtue ethics and critical theory, which opens up concerns for alienation. This fourth critique goes as follows: energy abundance might lead to alienation and, therefore, to dystopian versions of individual and social life. Karl Marx described four types of alienation, namely between the worker and the product of labor; between the worker and the labor; between people and nature; and between people in society [79]. Aligned with this taxonomy, abundance of energy can lead to alienation within a person, between people, and between people and nature. *Alienation within a person* can occur when an individual strives for energy abundance, because excess is a vice that does not lead to virtue and happiness. *Alienation among people* can occur as (energy) technologies mediate the relations between different actors and the world, including social relations among people [80, 81]. Technologies can also replace social interactions [82], or transform them in undesirable ways [83]. Finally, energy abundance can thwart relations *between people and nature* because of the ignorance concerning the energy resources’ origins in the natural ecosystems. People-nature alienation may worsen when human technological innovations (which require material and energy inputs) replace existing natural processes that provide essential ecological services, as is the case for biomimicry. We acknowledge that this initial exploration of the link between abundance and alienation, deserves and requires further deeper, broader and more robust engagement.

### The ambiguity of energy sufficiency

Energy utopias of sufficiency seem more in line with current planetary realities and thus counter the planetary boundaries and justice critiques often made to abundance ideals. In the current policy landscape, two major caveats hinder the uptake of energy sufficiency. Energy utopias of sufficiency offer an alternative conceptual basis to develop energy systems that remain within the constraints of planetary boundaries and at the same time prioritize justice concerns. In the current policy landscape, two major caveats hinder the uptake of energy sufficiency.

First, futures of energy sufficiency seem to be less attractive, especially today in light of the fast-paced progress of high energy-demanding digital technologies, such as cryptocurrencies and Artificial Intelligence. Even in *The New Atlantis*, when pollution has led to environmental collapse, people still dream of renewable energy abundance. Moreover, in both Le Guin’s novels, sufficiency only becomes an ideal after energy abundance led to collapse, in other words, out of necessity, and not because it was actively pursued as a goal. Degrowth futures in which we fly and consume less are not welcomed by many and are often met with resistance as they threaten the status quo. As such, there is a real danger that if energy sufficiency were to be promoted by governments on a large scale, citizens would protest and slow down the energy transition.

Second, embedding energy sufficiency in energy policies and technologies inevitably runs into practical and philosophical problems because, in reality, the lines between energy sufficiency and abundance are often contextual and situated on a sliding scale rather than corresponding to specific universal thresholds. Indeed, sufficiency is a relational concept that depends on both spatial-temporal contexts, demographics, and social practices. For example, different geographical contexts influence the amount of energy needed to sustain basic needs. Energy supply and access are often essential for meeting various human needs, including food security, protection from natural disasters, healthcare, mobility, and so on. As a result, a mild climate requires less energy for heating or air conditioning than more extreme climates. In a similar vein, individual characteristics such as age, sex, and disabilities determine the amount of energy that is required [84].

Moreover, the ‘need’ for energy is determined to a great extent by social practices. Inspired by Schatzki, Shove and Walker argue that energy is not used for its own sake, but to accomplish social practices such as cooking, transport, heating, and online meetings [5]. As a result, they claim that “energy demand is consequently dynamic, social, cultural, political and historical: it is bound up with the temporal rhythm of society and with what people do” [5]. Additionally, they argue that material arrangements, including energy infrastructures, create new social practices, and these social practices, in turn, reproduce, legitimize, and transform material arrangements. For example, they describe how “attempts to redefine a range of everyday practices such that electricity became a normal and necessary part of doing things like lighting, cooking and heating. It is plainly obvious that without moves of this kind there would be no ‘need’ for electricity at all” [5]. Moreover, Walker et al. describe how air conditioning becomes “needed” in a hospital in the UK through a complex network of social practices and material arrangements [85]. As a result of this co-constitutive relation between material arrangements and social practices, “societies are increasingly dependent on reliable supplies of electricity and oil in particular” [5]. The global energy demand has increased dramatically over the past few hundred years as a consequence of co-constitutive social practices and technological developments, and what used to be enough is now no longer sufficient.

In this light, it becomes unclear which social practices are essential and which are superfluous. As such, striving for energy sufficiency implies a thorough reflection on what and whose needs *really* deserve fulfilment. Several philosophers may be of assistance in navigating his question. Philosophers Epicurus already distinguished between *natural and necessary desires*, *natural but unnecessary desires*, and *empty desires* that can never be satisfied such as immortality, wealth, and fame [86]. The original ethical theory of Epicureanism prescribes merely fulfilling the natural and necessary desires as they lead to freedom from unnecessary desires, resulting in true pleasure [Vatican Sayings 77, described in 66]. The good life is not one in which all desires can be fulfilled by an unlimited amount of resources; only some desires are worth fulfilling. Besides Epicurus, many other accounts of vital human needs have been given throughout the history of philosophy. For example, Powers and Faden [88] understand universal human needs as *well-being*, including health, knowledge and understanding, personal security, equal respect, personal attachments, and self-determination. Another alternative is provided by Sen and Nussbaum’s notion of human capabilities, which represent conditions for a good and dignified life [89, 90].

It is possible to embed energy sufficiency in energy policies, however, people in different societies with different social practices will inevitably “need” different amounts of energy. In other words, uncertainty about where the demarcation between “needs” and “wants” lies does not imply that it is always impossible to establish when something is truly needed. This raises significant justice questions: Can countries with high energy needs justify their consumption levels by referring to the status quo (i.e., already existing social practices that require significant amounts of energy)? What would this mean for the allocation of future CO2-emission budgets and effort-sharing for climate mitigation among countries [91]? To what extent do different needs justify an unequal distribution of energy among and within different peoples and countries? In sum, embedding ideals of energy sufficiency in energy and climate policies will inevitably run into practical as well as philosophical, in particular normative ethical problems.

### Outlook: what future should we strive for?

From the normative energy ethics reflection above, we conclude that renewable energy utopias of abundance can motivate stakeholders towards adopting various climate-friendly behaviours, which is a significant benefit. However, one should not forget the existence of several ethical problems related to energy abundance as a desired future. Although renewable energy sufficiency mitigates the disadvantages of striving for energy abundance, it faces two practical problems that hinder the embedding of this ideal in energy policies. As such, there is an ethical dilemma: should we, in our climate and energy policies, technologies, and energy transitions in general, strive for renewable energy sufficiency or abundance?

Instead of trying to fully reconcile the tension, we return to one of the outcomes of these science fiction narratives, which invite readers to consider distinctions between what energy futures we, as a society, want and what energy futures we should strive for in energy policies and innovations. Most people may desire energy abundance, and as it might motivate action towards large-scale renewable energy, this may be a meaningful strategic and motivational approach. However, this does not imply that energy abundance is to be embedded uncritically in energy policies and technological agendas. Considering the critiques of energy abundance, one may conclude that we should collectively strive for energy sufficiency instead. This implies that the second concern around energy sufficiency, namely its ambiguity and related philosophical questions, should be tackled head-on, instead of avoided, which requires interdisciplinary collaborations between energy philosophy and energy social sciences. Rather than simply shifting towards sufficiency, we invite energy planners, policymakers, and scholars of energy futures to be aware of the distinctions and to be more attentive to how assumptions of abundance and sufficiency are embedded into their visions of energy futures.

## Conclusions

In this paper, we distinguished between two types of desired energy futures, namely renewable energy sufficiency - envisioning a world in which only those needs that truly make people happy are fulfilled, implying an ecologically balanced lifestyle – and renewable energy abundance, in which an endless energy supply fulfils all wants and needs. These categories are meaningful for identifying and understanding implicit and explicit assumptions on desired futures embedded in technological innovations, climate policies, and in our own minds as citizens and energy consumers. Identifying and understanding normative assumptions about the future pave the way towards critical ethical reflection. Such a reflection is crucial because the dominant desired futures become embedded in energy technologies and policies, thus steering energy consumption and energy transitions.

We tapped into Sci-Fi and normative energy ethics perspectives to identify critical views on renewable energy abundance and sufficiency. Sci-Fi describes a broad array of energy futures while painting them as more or less utopian or dystopian, enabling critical assessment. We then turned to normative energy ethics to flesh out the critiques by tapping into theories on the good life, justice, and human needs. We found that both Sci-Fi and various energy ethics perspectives highlight important limits and dangers to utopias of renewable energy abundance, varying from crossing planetary limits to erroneously pursuing dystopian visions of the good life. However, at the same time, ideas of renewable energy abundance seem strong drivers for the energy transition, especially in contexts of fast-paced progress of digital technologies such as cryptocurrencies and Artificial Intelligence, while ideas of energy sufficiency are unappealing as they seem to threaten the status quo. Much more future work can be dedicated to navigating the philosophical and normative ethical questions around renewable energy sufficiency as ideals in energy policymaking. The paper showcases how both Sci-Fi and normative energy ethics are instrumental to critical thinking on assumptions of desired futures embedded in climate policies and socio-technical energy systems. Such critical reflections are crucial, as ideals of abundance and sufficiency continue to shape renewable energy transitions.

## Data Availability

Data sharing is not applicable to this article as no datasets were generated or analysed during the current study.

## Footnotes

1 We maintain that abundance is a synonym of “inexhaustible” or “plentiful” while “endlessness” or “infiniteness” is ”indefinably large”, “ countlessly great”, “immense”. This means that the latter expressions are sufficient yet not necessary conditions for abundance, which represents a level of availability that is even harder or impossible to measure or use.
